# Supplementary material for: Physiological and Genomic Characterization of a Novel Obligately Chemolithoautotrophic, Sulfur-Oxidizing Bacterium of Genus Thiomicrorhabdus Isolated from a Coastal Sediment
Source: Microorganisms. 2023 Oct 15;11(10):2569. doi: 10.3390/microorganisms11102569 (PMC10608990; doi:10.3390/microorganisms11102569)
Supplement: Supplementary file 1 [file microorganisms-11-02569-s001.zip › microorganisms-2624246-supplementary.pdf]

microorganisms

Supplements

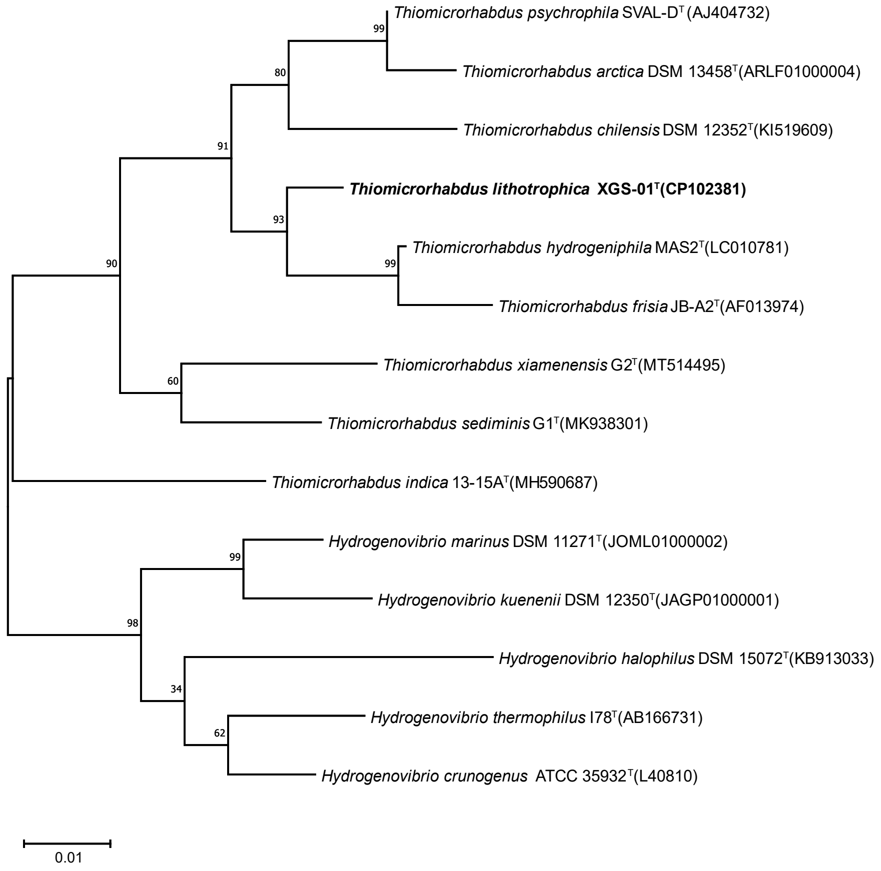

Figure S1. Maximum-likelihood phylogenetic tree based on 16S rRNA gene sequences showing the relationship between strain XGS-01T and members of the genus *Thiomicrorhabdus*. Bootstrap values (>50%) based on 1000 replicates are shown at branch nodes. Bar, 0.01 substitutions per nucleotide position.

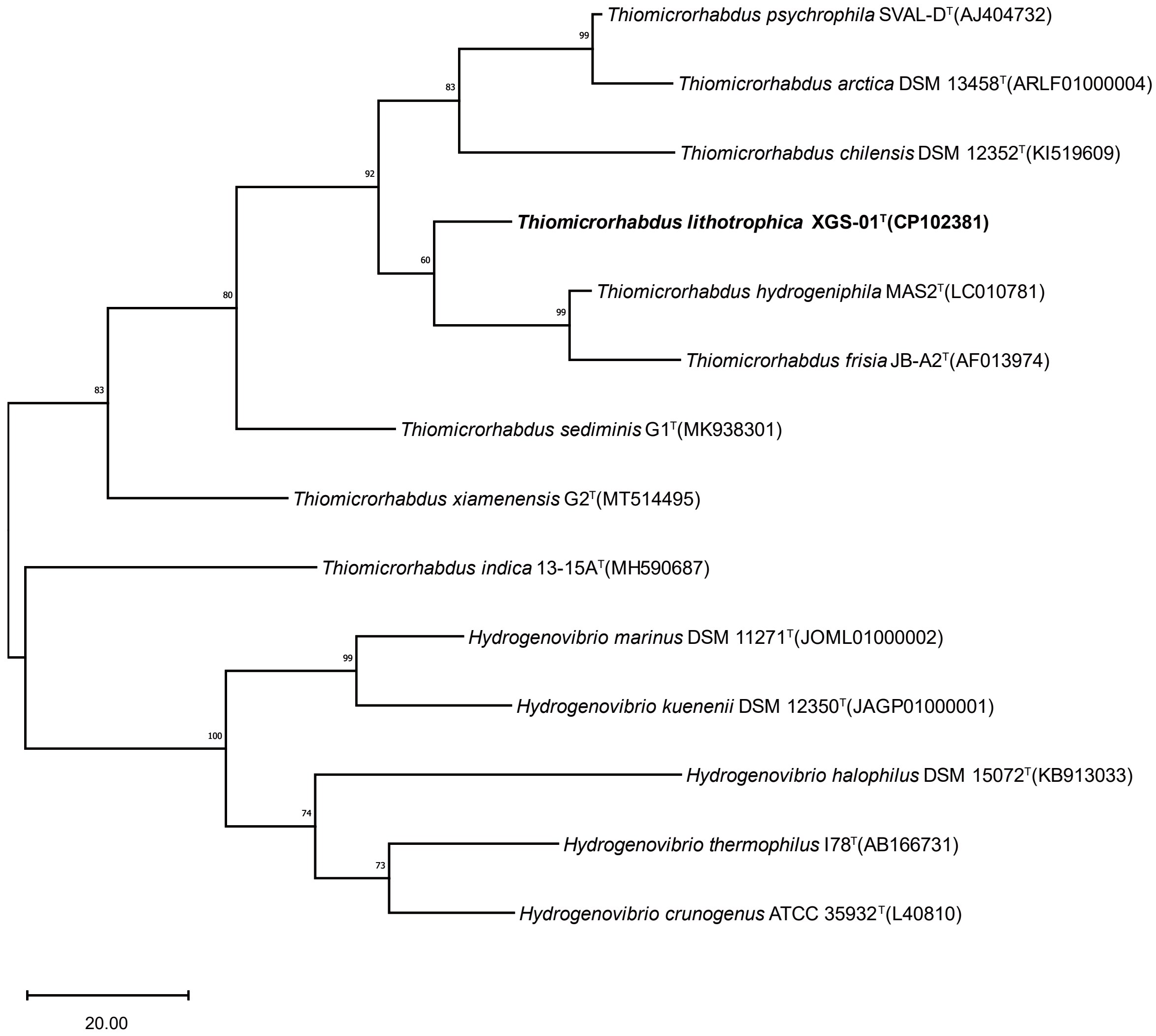

Figure S2. Maximum-Parsimony phylogenetic tree based on 16S rRNA gene sequences showing the relationship between strain XGS-01T and members of the genus *Thiomicrorhabdus*. Bootstrap values (>50%) based on 1000 replicates are shown at branch nodes. Bar, 0.01 substitutions per nucleotide position.

> *Thiomicrorhabdus lithotrophica* XGS-01^{T} 16S ribosomal RNA gene, complete sequence

ATTAAACTGAAGAGTTTGATCCTGGCTCAGAATGAACGCTGGCGGTAGGCTTAACACATG
CAAGTCGGACGGAAACGATAGAGAAACTTGTTTTTCTAGGCGTCGAGTGGCGGACGGGTG
AGTAACGCGTGGGAATCTACCCTGTAGTTGGGGACAACATATGGAAACGTATGCTAATAC
CGAATATGCTCTACGGAGTAAAGGAGCCCTCTTCTTGAAAGGTTTCGCTATAGGATGAGC
CCGCGTGAGATTAGCTAGTTGGTAAGGTAATGGCTTACCAAGGCGACGATCTCTAGCTGG
TTTGAGAGGATGATCAGCCACACTGGGACTGAGACACGGCCCAGACTCCTACGGGAGGC
AGCAGTGGGGAATATTGCACAATGAGCGAAAGCTTGATGCAGCCATACCGCGTGTGTGA
AGAAGGCCCGAGGGTTGTAAAGCACTTTCAATTGTGAGGAAGATAGAGTAGTTAATACCT
GCTTTATTTGACGTTAACTTTAGAAGAAGCACCGGCTAACTCTGTGCCAGCAGCCGCGGTA
ATACAGAGGGTGCAAGCGTTATTCGGAATTACTGGGCGTAAAGCGCGCGTAGGCGGGCT
ATTAAGTCAGTTGTGAAAGCCCTGGGCTCAACCTGGGAACTGCATCTGATACTGGTAGTCT
AGAGTTTAAGAGAGGGAAGTGGAATTCCAGGTGTAGCAGTGAAATGCGTAGATATCTGG
AGGAACATCAGTGGCGAAGGCGACTTCCTGGCTTAAAACTGACGCTGAGGTGCGAAAGC
GTGGGTAGCGAACGGGATTAGATACCCCGGTAGTCCACGCCGTAAACGATGTCAACTAGT
TGTTGGTCTTATTAAAAAGATTAGTAACGAAGCTAACGCGATAAGTTGACCGCCTGGGGA
GTACGGTCGCAAGATTAAAACTCAAAGGAATTGACGGGGGCCCGCACAAGCGGTGGAGC
ATGTGGTTTAATTCGATGCAACGCGAAGAACCTTACCATCCCTTGACATGTAGAGAACTTT
CTAGAGATAGATTGGTGCCTTCGGGAACTCTAACACAGGTGCTGCATGGCTGTCGTCAGCT
CGTGTCGTGTGAGATGTTGGGTTAAGTCCCGCAACGAGCGCAACCCTTATCATTAGTTGCT
AACATTTAGTTGAGAACTCTAATGAGACTGCCGGTGATAAACCGGAGGAAGGCGGGGAC
GACGTCAAGTCATCATGGCCCTTATGGGATGGGCTACACACGTGCTACAATGGGGGGTAC
AAAGAGCAGCCAACTGGCAACAGTGCGCGAATCTCAAAAAACCCCTCGTAGTCCGGATT
GGAGTCTGCAACTCGACTCCATGAAGTCGGAATCGCTAGTAATCGCGGATCAGAATGCCG
CGGTGAATACGTTCCCGGGCCTTGTACACACCGCCCGTCACACCATGGGAGTGGATTGCA
AAAGAAGTAGGTAGCTTAACCTTCGGGAGAGCGCTTACCACTTTGTGGTTCATGACTGGG
GTGAAGTCGTAACAAGGTAGCCCTAGCGGAAGCTGGGGCTGGATCACCTCCTTTAA
